# Redox Homeostasis in Well-differentiated Primary Human Nasal Epithelial Cells

**DOI:** 10.33696/signaling.3.083

**Published:** 2022

**Authors:** Ayaho Yamamoto, Peter D. Sly, Anna Henningham, Nelufa Begum, Abrey J. Yeo, Emmanuelle Fantino

**Affiliations:** 1 Child Health Research Centre, The University of Queensland, South Brisbane, Queensland 4101, Australia; 2 The University of Queensland Centre for Clinical Research, Herston, Queensland 4029, Australia

**Keywords:** Oxidative stress, Airway epithelium, Air-liquid interface culture, Signaling pathways, Mitochondria

## Abstract

Oxidative stress (OS) in the airway epithelium is associated with inflammation, cell damage, and mitochondrial dysfunction that may initiate or worsen respiratory disease. Redox regulation maintains the equilibrium of pro-oxidant/antioxidant reactions but can be disturbed by environmental exposures. The mechanism(s) underlying the induction and impact of OS on airway epithelium and how these influences on respiratory disease is poorly understood. The aim of this study was to develop a stress response model in primary human nasal epithelial cells (NECs) grown at the air-liquid interface (ALI) into a well-differentiated epithelium and to use this model to investigate the mechanisms underlying OS. Hydrogen peroxide (H_2_O_2_) was used to induce acute OS and the responses were measured with trans epithelial electrical resistance (TEER), membrane permeability, cell death (LDH release), mitochondrial reactive oxygen species (mtROS) generation, redox status (GSH/GSSG ratio), cellular ATP, and signaling pathways (SIRT1, FOXO3, p53, p21, PINK1, PARKIN, NRF2). Following 25 mM (sensitive) or 50mM (resistant) H_2_O_2_ exposure, cell integrity decreased (*p*<0.05), GSH/GSSG ratio reduced (*p*<0.05), and ATP production declined by 83% (*p*<0.05) in the sensitive and 55% (*p*<0.05) in the resistant group; mtROS production increased 3.4-fold (*p*<0.001). Significant inter-individual differences between healthy humans with regards to susceptibility to OS, and differential activation of various pathways (FOXO3, PARKIN) were observed. These intra-individual differences in susceptibility to OS may be attributed to resistant individuals having more mitochondria or greater mitochondrial function.

## Introduction

Oxidative stress (OS) arises when there is an imbalance between oxidative stressors and the ability of cells to detoxify oxidants or to repair oxidant-induced damage [1]. Oxidants can be generated from endogenous or exogenous sources. An endogenous source is respiration in mitochondrial electron transport; exogenous sources include environmental stressors [2]. The overall oxidant – antioxidant status is mediated and counteracted by enzymatic and non-enzymatic antioxidant defense systems including superoxide dismutase (SOD), catalase (CAT) and glutathione peroxidase (GPx) to maintain physiological homeostasis [3]. The oxidation-reduction (redox) reactions are regulated by redox signaling and redox control. The maintenance of a redox balance is named redox homeostasis [4].

Since the respiratory epithelium is directly exposed to the ambient environment, they are at high risk of OS. Antioxidant defense and OS play a critical role in various respiratory disorders such as asthma, chronic obstructive pulmonary disease (COPD), idiopathic pulmonary fibrosis (IPF), ataxia-telangiectasia (A-T) and respiratory infections. COPD is the third most common cause of death worldwide. The main risk factors of COPD are tobacco smoking and air pollution [5]. COPD is linked to increased mitochondrial reactive oxygen species (mtROS) production, reduced numbers of mitochondria and decreased intracellular antioxidants [6]. High OS levels were recorded in the lung and the breath of patients with COPD, partially explained by the multiple oxidants in cigarette smoke, but also results from release of oxidants from activated inflammatory cells such as neutrophils and macrophages. Even when patients stopped smoking OS persists [7].

Mitochondria are not only a powerhouse within the cell, but also involved in cell survival [8]. Most of the cellular reactive oxygen species (ROS) are generated by mitochondria. The main contributor to the oxidative damage is hydrogen peroxide (H_2_O_2_), which leaks from mitochondria. The accumulation of oxidative damage may lead to mitochondrial dysfunction [9]. Mitochondrial homeostasis involves the mitochondrial redox system, mitophagy, biogenesis and oxidative phosphorylation to maintain the health of mitochondria. Cellular homeostasis is also regulated through the membrane potential (ΔΨm), producing acetyl coenzyme A by mitophagy. Mitochondrial function can be disrupted by multiple forms of endogenous and exogenous stress, which trigger an inflammatory response, increasing ROS production, and decreasing ΔΨm [9]. Mitophagy is a selective degradation of damaged mitochondria. Mitochondrial fission and fusion play the main role of selecting mitochondria for mitophagy. Substantial loss of ΔΨm promotes fission leading to mitophagy which may result in removal of excess mtROS, mitochondrial DNA (mtDNA) and other associated factors. The mitochondrial stress markers PINK1 and PARKIN are increased in patients with COPD [9]. Compared with wild-type mice, both *Pink1* and *Parkin* knockout (KO) mice showed an increase in ROS levels and a decline in mitochondria function. Thus, Pink1 and Parkin seems to play an important role in removing mtROS [10,11].

OS activates multiple genes including sirtuin family members (SIRTs), they are closely associated with mitochondrial integrity, stress tolerance mediation and inflammation [8]. SIRT1 is an NAD^+^-dependent protein deacetylase which is involved in multiple molecular pathways including stress resistance, inflammation, DNA repair, apoptosis, mitochondrial regulation, and longevity [12]. One of key functions of SIRT1 is modulating mitochondrial biogenesis and functions through nuclear to mitochondrial signaling pathways [13]. SIRT1 can be activated by OS and plays an important role in regulating multiple molecular pathways including stress resistance, forkhead box O3 (FOXO3) and p53 [14]; and antioxidant responses, nuclear factor erythroid 2-related factor 2 (NRF2) [15].

The airway epithelium provides the first line of innate immune protection to defend against environmental stressors and infections [16]. OS in the airway epithelium induces inflammation, cell damage, and mitochondrial dysfunction that may initiate or worsen respiratory diseases such as acute lung injury, asthma, and COPD [17,18]. Appropriate cellular and mitochondrial stress responses are critical for maintaining tissue homeostasis, integrity, and function. The mechanism underlining the induction and impact of OS on airway epithelium remains unclear.

In the present study, we collected primary human nasal epithelial cells, grew them into well-differentiated epithelium in air-liquid interface (ALI) cultures, and used this model to investigate the effect of OS on airway epithelium as it provides a physiological relevant model of the human upper airways [19]. The nose is the point of first contact with environmental exposures, it is therefore important to understand the impact of OS on the nasal epithelium.

## Materials and Methods

### Cell collection

Healthy non-atopic, non-smoking adult volunteers between the ages of 18 and 65 years were recruited, and primary human nasal epithelial cells (NECs) were collected using Rhino-Pro Nasal Curette (Arlington Scientific, UT, USA). (Ethics approval: No.#UQ2017000520; HREC61894; UQ2020001742).

The nasal scrapings were taken from the inferior turbinate in each nostril by gently pressing the cupped tip on mucosal surface and moving outward to collect sample [20]. Cells were then seeded onto collagen coated 24-well plates. Cells were grown as submerged cultures in PneumaCult^™^-Ex Medium (STEMCELL Technologies, BC, Canada) for approximately two weeks until they reached passage 2 and were cryopreserved in FBS with 10% DMSO.

### Air-liquid interface culture

Cells were seeded (4*10^4^ cells/insert) onto 6.5 mm Transwell^®^ with 0.4 μm Pore Polyester Membrane Inserts (Corning, NY, USA) in 24 well plates. After approximately three days of cell division, the cells were “air-lifted”, a process which involved removing media from the apical chamber and replacing the media in the basal chamber with PneumaCult-ALI Medium (STEMCELL Technologies, BC, Canada). Cells were maintained in ALI conditions for at least three weeks until beating cilia were observed under light microscopy and a high trans epithelial electrical resistance (TEER) was established. A successfully differentiated ALI culture contains basal cells, tight junctions, secretory cells (primarily mucus-secreting goblet cells) and ciliated epithelial cells (Figure S1).

### Establishing a stress response model

The oxidant H_2_O_2_ was used to induce OS in the airway epithelium. Fully differentiated NECs were exposed to various concentrations of H_2_O_2_ (0, 10, 25, 50, 100 mM) in Hanks’ Balanced Salt solution (HBSS; Sigma-Aldrich, MI, USA) in the apical chamber for one hour as an acute exposure.

### Trans-epithelial electric resistance

TEER is a widely accepted quantitative measurement to examine the integrity of tight junction dynamics in epithelial cell culture at ALI and can be performed in real-time without causing cell damage [21]. TEER value was measured using the EVOM2^™^ meter (World Precision Instruments, FL, USA) as per the manufacturers’ instructions at 0, 1, 4 and 24-hour time points relative to H_2_O_2_ exposure and the values were recorded as ohmic resistance.

### Epithelial Permeability

A permeability assay was performed as per the method of Turksen [22]. In short, a 100 μl volume of media with 0.5 mg/ml FITC-dextran was added to the apical chamber and incubated for 2 hours at 37°C with 5% CO_2_ at 0, 1, 4 and 24-hour time point relative to H_2_O_2_ exposure. The basal media was collected, and the fluorescence measured (excitation of 490 nm, emission of 520 nm) by CLARIOstar (BMG LABTECH, Offenburg, Germany) alongside FITC-dextran standards to calculate the amount of FITC-dextran that had migrated through the epithelium and the transwell during the incubation.

### Cell death

A LDH release assay (CytoTox 96^®^ Non-Radioactive Cytotoxicity Assay; Promega Corporation, WI, USA) was used to evaluate the level of cell damage or death. A 100 μl volume of HBSS was placed into the apical surface of differentiated epithelium for 10 minutes, and this HBSS was collected at 0, 1, 4 and 24-hour time point relative to H_2_O_2_ exposure and the amount of LDH secretion was quantified as per the manufacturer’s instructions.

### Mitochondrial ROS

Red mitochondrial superoxide indicator (MitoSOX^™^, Invitrogen^™^, CA, USA) was used to detect the generation of mtROS and the fluorescence was determined by Zeiss Confocal LSM 710 (ZEISS, Oberkochen, Germany). Briefly, a 100 μl volume of 5 μM MitoSOX in HBSS was added onto the transwell membranes and incubated in 37°C for one hour. Cells were fixed in 3.7% paraformaldehyde (PFA)/PBS for 10 minutes at room temperature and nuclei were stained with Hoechst 33342 (Invitrogen^™^, CA, USA). The membrane was then excised from the transwell insert and mounted directly on slide with 20 μl of ProLong^™^ Gold Antifade Mountant (Invitrogen^™^, CA, USA). Images were visualized and captured with confocal at 580 nm. Fluorescence intensity was subsequently quantitated with the ImageJ software (National Institutes of Health, Bethesda, MD) [23].

### Glutathione

The ratio of reduced glutathione (GSH) to oxidized glutathione (GSSG) (GSH/GSSG ratio) can change over the time after oxidant exposure, basal H_2_O_2_ (1 mM) exposure was performed in this assay.

Cells were lysed with mammalian cell lysis buffer and deproteinized as per manufacturer’s instruction (Abcam, Cambridge, UK). Samples were then diluted 1:10 in H_2_O and GSH and total glutathione were measured according to manufacturer’s instruction (GSH/GSSG Ratio Detection Assay Kit II; Abcam, Cambridge, UK). GSSG was calculated as: GSSG = (Total Glutathione – GSH)/2.

### ATP production

Cellular adenosine triphosphate (ATP) (Luminescent ATP Detection kit; Abcam, Cambridge, UK) was measured according to manufacturer’s instruction. Briefly, a 50 μl volume of detergent was added to each transwell and cells were scraped off from the transwell. Cell lysates were then transferred into microcentrifuge tubes and the tubes were shaken for 5 minutes in an orbital shaker at 700 rpm to lyse the cells and stabilize the ATP. Samples were diluted in H_2_O at 1:10 and transferred to a clear 96-well plate. 50 μl Substrate Solution was subsequently added to each well and the microplate was shaken for 5 minutes in an orbital shaker at 700 rpm. Luminescence was measured by CLARIOstar following 10 minutes incubation.

### RNA extraction and quantitative Reverse Transcription PCR (qRT-PCR)

At the 24-hour time point post H_2_O_2_ exposure, total RNA was isolated from cells using TRIzol^™^ reagent (Invitrogen^™^, CA, USA) and RNeasy Mini Kit (Qiagen, Hilden, Germany). A total of 100 ng RNA from each sample was reverse-transcribed into cDNA using a High Capacity cDNA Reverse Transcription Kit (Applied Biosystems^™^, CA, USA). qPCR was performed with Taqman primers (Applied Biosystems^™^, CA, USA): *SIRT1* (Hs01009005_m1), *FOXO3* (Hs00818121_m1), *CDKN1A* (*p21*; Hs99999142_m1), *TP53* (*p53*; Hs01034249_m1), and *PINK1* (Hs00260868_m1) with FAM dye and housekeeping gene Eukaryotic 18S rRNA Endogenous Control (4319413E) with VIC dye.

Quantification of gene expression was performed using a ViiA^™^ 7 Real-Time PCR System (Applied Biosystems, MA, USA). The running conditions were as follow: 50°C for 2mins, 95°C for 20sec, followed by 40 cycles of 95°C 1 sec and 60°C 20 sec. The relative mRNA expression levels were normalized to 18s using the 2^−ΔΔCq^ methods.

### Western blot

Cells were lysed with a 100 μl of cold 2% sodium dodecyl sulfate (SDS)/PBS lysis buffer containing cOmplete^™^, Mini Protease Inhibitor Cocktail and PhosSTOP^™^, phosphatase inhibitor (Sigma-Aldrich, MI, USA). Protein concentration was measured by Pierce^™^ BCA Protein Assay Kit (Thermo Fisher Scientific, MA, USA). Samples were mixed with LDS sample buffer and sample reducing agent (Invitrogen^™^, CA, USA), and incubated at 70°C for 10 minutes.

Equal quantities of protein were loaded to Bolt^™^ 4 to 12%, Bis-Tris Gel (Invitrogen^™^, CA, USA) and electrophoresed at 200V for 30 minutes. The gel was then transferred to Immobilon-P PVDF membrane (Merck KGaA, Darmstadt, Germany). Membranes were blocked in blocking buffer; 3% BSA (phosphorylated protein and PARKIN), 5% (w/v) nonfat dry milk (SIRT1) or 5% BSA (GAPDH) in tris-buffered saline with Tween 20 (TBST) for one hour at room temperature and subsequently incubated with primary antibodies, SIRT1 (1:1000) (8469S; Cell Signaling Technology, MA, USA), phosphorylated-SIRT1 (1:1000) (2314S; Cell Signaling Technology), phosphorylated-PARKIN (1:1000) (PA1-4735; Invitrogen^™^), GAPDH (1:1000) (2118S; Cell Signaling Technology); PARKIN (1:500) (39-0900; Invitrogen^™^) in blocking buffer at 4°C overnight. The following day, membranes were incubated with appropriate fluorescent secondary antibodies, anti-rabbit (1:10,000) (5366P; Cell Signaling Technology) or mouse (1:5,000) (5257P; Cell Signaling Technology) at room temperature for one hour. The results were detected using the LI-COR Odyssey (BioAgilytix, NC, USA) and quantified by ImageJ software.

### NRF2 nuclear translocation assay

NRF2 nuclear translocation was examined by immunostaining as described previously [24]. In short, cells were fixed in 3.7% PFA/PBS for 10 minutes at room temperature and were permeabilized using 100 μl of 0.5% Triton X100/PBS for 10 minutes and blocked using 200μl of blocking buffer (2% bovine serum albumin (BSA), 0.2% Triton X100, PBS) for one hour at room temperature. A 100 μl of NRF2 antibody (sc-365949; Santa Cruz Biotechnology, TX, USA) in blocking buffer (1:100 dilution) was subsequently applied and incubated for four hours at room temperature and 4°C overnight. The next day, a 100 μl of anti-mouse secondary antibody, Alexa Fluor 647 (A21235; Invitrogen^™^, CA, USA) in blocking buffer (1:250 dilution) was applied and incubated for one hour at 37°C in a humidified incubator. Cells were then washed three times with PBS for three minutes and a 100 μl of Hoechst 33342 nuclei stain in PBS (1:10,000 dilution) was applied for 10 minutes. The transwell membrane containing the cells was excised from the insert using No.11 scalpel blade in a clockwise motion and mounted directly on a glass slide. 20 μl of ProLong^™^ Gold Antifade Mountant (Invitrogen^™^, CA, USA) was applied, and coverslip was placed gently on top. Images were visualized and captured on the Zeiss Confocal LSM 710 and the mean intensity of nuclear NRF2 was quantified by the Image J software.

### Statistical analysis

All the graphs were plotted using GraphPad Prism 9.00 (GraphPad, CA, USA) and were expressed as the mean ± standard error of the mean (SEM).

Kruskal-Wallis rank test with Dunn’s multiple-comparison test was performed to compare different conditions. Two-sample Wilcoxon rank-sum (Mann-Whitney) test was used to compare sensitive and resistant groups. *P*<0.05 was considered to indicate a statistically significant difference.

## Results

### H_2_O_2_ exposure reduces epithelial barrier integrity

**Figure 1.**
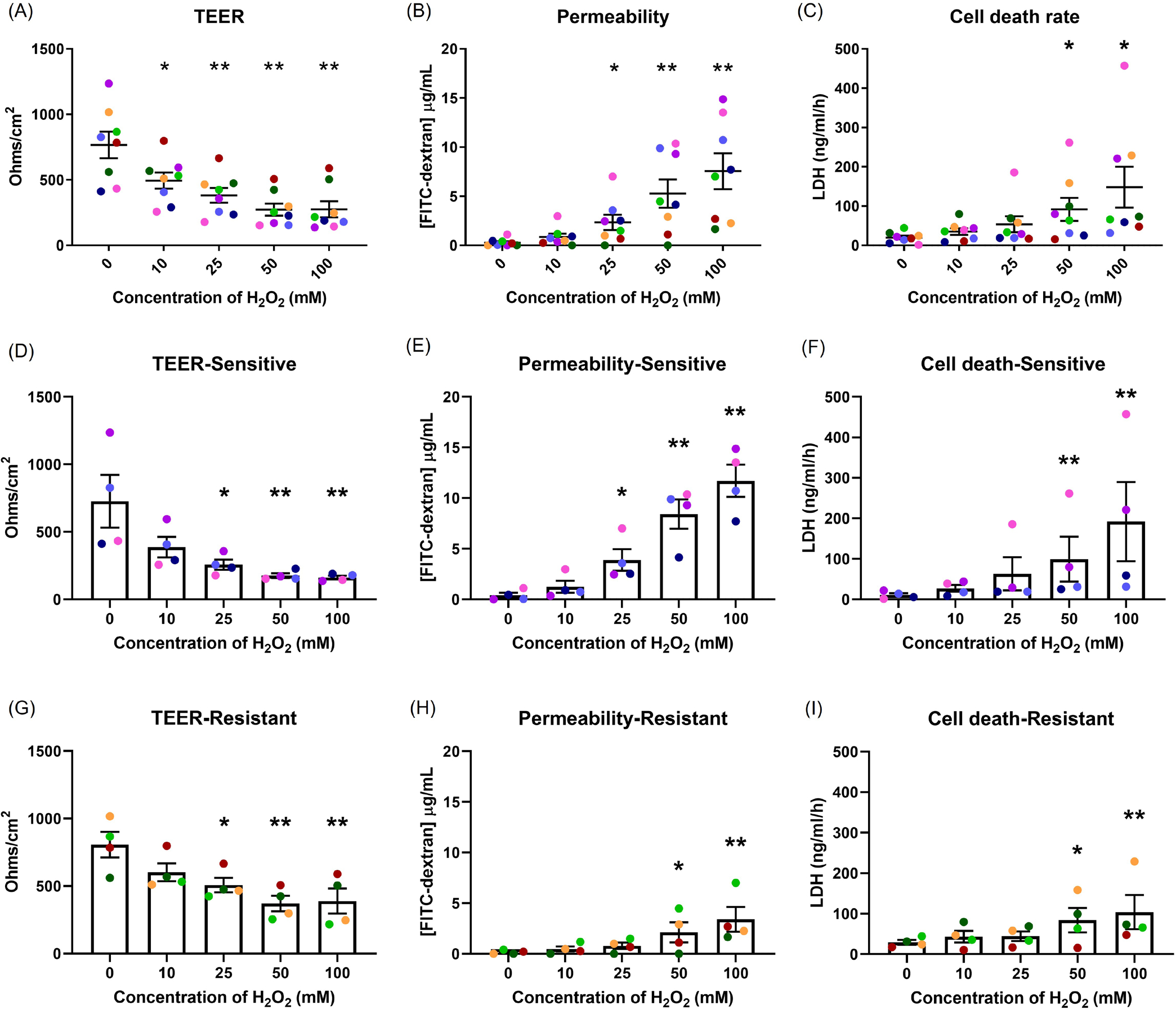
The effects of oxidant exposure on epithelial integrity. Well-differentiated NECs were exposed to different concentrations of H_2_O_2_, and the effects measured by TEER **(A)**, permeability to FITC-dextran **(B)** and cell death, indicated by LDH secretion **(C)**. Each concentration group was compared to control. There was a dose-dependent effect on epithelial integrity. Differences were seen in the responses in TEER **(D, G)** and permeability **(E, H)** between the sensitive and resistant groups, but not in cell death **(F, I)**. Data presented as mean ± SEM (n=8; *p<0.05; ** *p*<0.01, compared with control). A single color was used in all figures to represent a single donor.

Epithelial integrity was examined following H_2_O_2_ exposure (Figure 1). A dose-dependent decrease in TEER with increasing H_2_O_2_ concentration was observed (Figure 1A). Similarly, with an increase in H_2_O_2_ concentration, a dose-dependent increase in permeability and cell death rate occurred (Figures 1B and 1C). Differences in sensitivity to H_2_O_2_ between donors were observed.

To examine the differences in response, cells from 12 healthy donors were grown and differentiated at ALI. Following 50 mM H_2_O_2_ exposure, TEER and permeability assay were performed. A great variability between donors was observed (Figures S2A and 2B). Therefore, different concentrations of H_2_O_2_ were tested in each donor and the concentration required to achieve a comparable baseline response was reported. The permeability assay was used to determine the comparable baseline (Figure S2D). It was an interesting finding that one dose can be harmful for some donors while not for others. For example, 300 mM H_2_O_2_ exposure on Donor 3 had a similar effect to 25 mM H_2_O_2_ exposure on Donor 1. This means that a high dose of H_2_O_2_ can cause extreme damage to cells obtained from Donor 1 but not Donor 3. On the other hand, a low dose of H_2_O_2_ that did not affect Donor 3, was sufficient to cause damage in Donor 1. There was a 12-fold difference in H_2_O_2_ concentrations used to achieve a comparable baseline between Donor 3 and Donor 1.

Donors were then grouped into two groups, sensitive or resistant, according to the dose of H_2_O_2_ required to cause an increase in FITC-dextran concentration to greater than 10 μg/ml (Figures S2D & S3). Subjects in whom a concentration of H_2_O_2_ of ≤ 25 mM caused an increase in FITC-dextran concentration to >10 μg/ml were considered as sensitive to OS. Those that required >25 mM H_2_O_2_ to induce an increase in FITC-dextran concentration to >10 μg/ml were considered as resistant to OS. These groupings were used for subsequent examinations reported in this study as similar responses were observed within the group in multiple exposures and different measurements. Different concentrations of H_2_O_2_ were used in each group to achieve a comparable baseline response, with 25 mM H_2_O_2_ used in the sensitive group and 50 mM H_2_O_2_ in the resistant group. These concentrations were sublethal in both groups and allowed us to investigate the cellular responses. A single color was used in all figures to represent a single donor throughout the results in all sections. No differences in donor background between the groups were observed (Table S1).

Those in the sensitive group had lower TEER (*p*=0.001) and increased permeability (*p*=0.006) compared with those in the resistant group following 25 mM, 50 mM and 100 mM H_2_O_2_ exposure (Figures 1D, 1E, 1G, 1H). The overall differences in cell death were not statistically different, however, 100 mM H_2_O_2_ caused more cell death in the sensitive group than in the resistant group (*p*=0.301; Figures 1F, and 1I).

### H_2_O_2_ exposure induced mitochondrial reactive oxygen species generation

**Figure 2.**
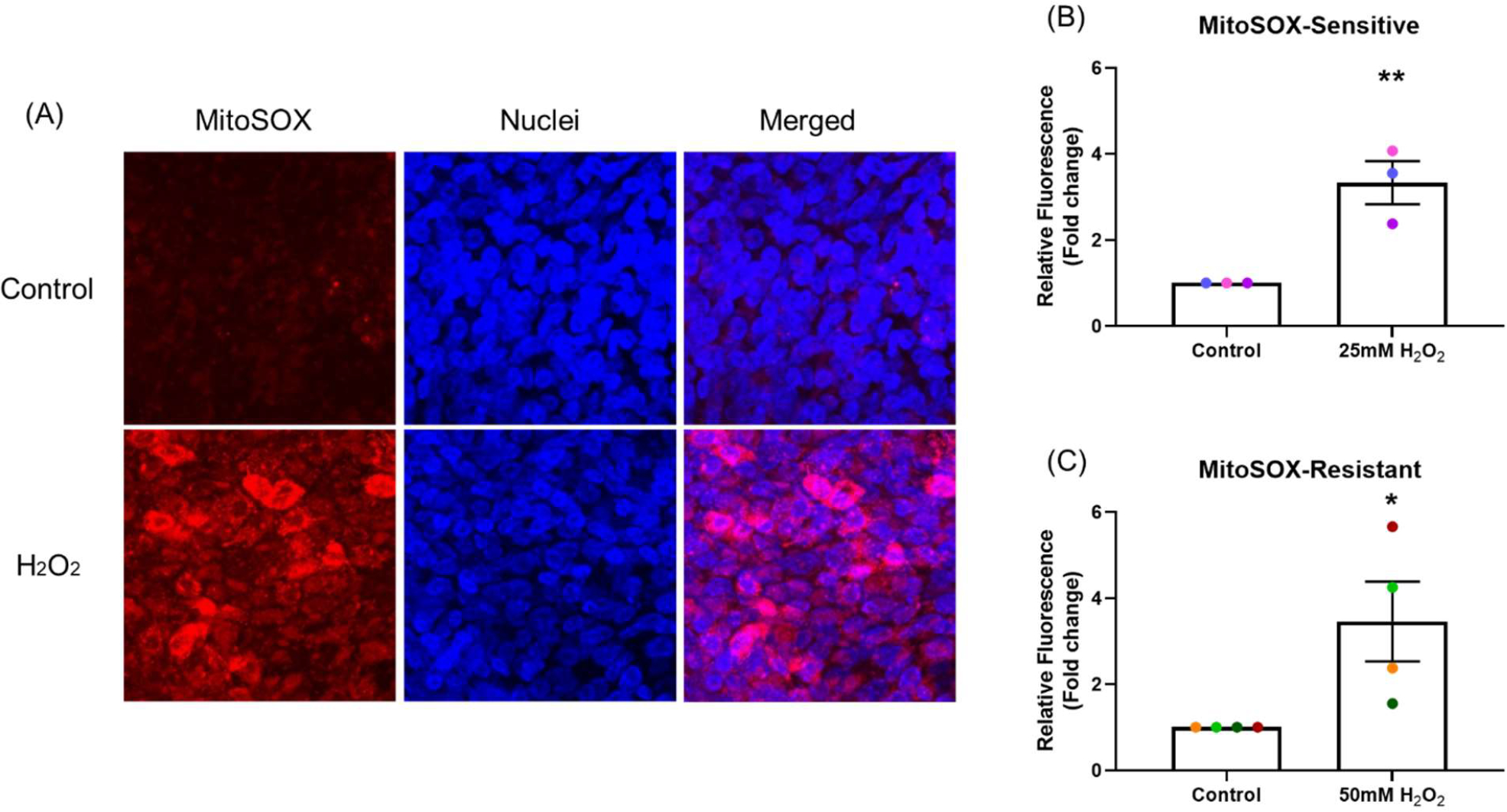
The effect of oxidant exposure on mitochondrial ROS production. After exposure to H_2_O_2_, mtROS generation was measured by MitoSOX and images captured by confocal microscopy, images are shown from representative sensitive donor (Red: mtROS; blue: nuclei). **(A)** mtROS can be seen in the cytoplasm (merged panel in A). Compared with control, mtROS production increased in NECs exposed to H_2_O_2_ in both sensitive **(B)** and resistant **(C)** groups. Data presented as mean ± SEM (n=7; *p<0.05; ** *p*<0.001, compared with control). A single color was used in all figures to represent a single donor.

As most of the intra-cellular ROS are generated by mitochondria (mtROS), mitochondrial homeostasis is critical for maintaining cell health. Increase in mtROS can cause mitochondrial dysfunction and lead to disease [17]. One hour after H_2_O_2_ exposure, mtROS production was significantly increased by 3.3-fold in the sensitive (*p*<0.001; Figure 2B) and 3.5-fold in the resistant donors (*p*<0.05; Figure 2C) (MitoSOX assay) compared with control (Figure 2).

### H_2_O_2_ exposure caused a decrease in GSH/GSSG ratio

**Figure 3.**
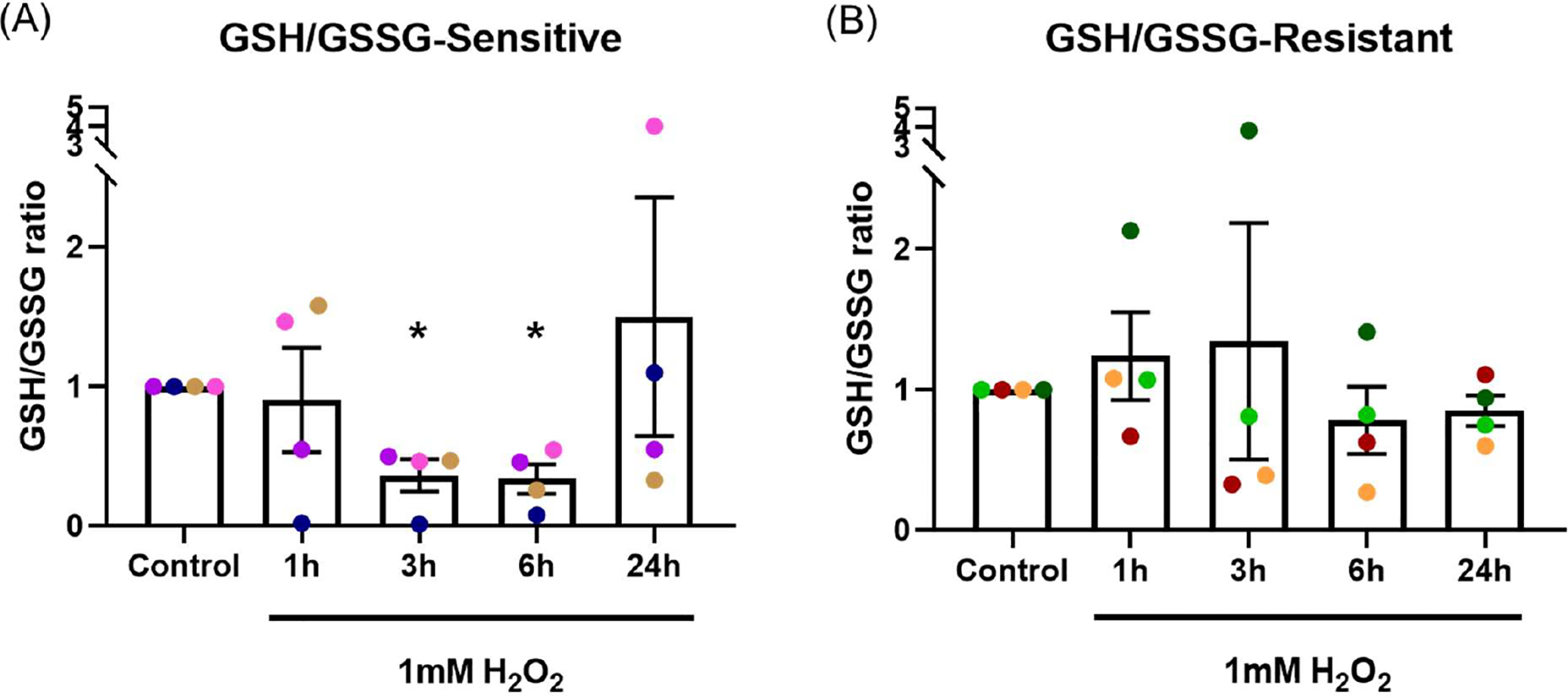
The effect of oxidant exposure on epithelial GSH/GSSG ratio. Following 1 mM H_2_O_2_ exposure in the basal chamber, GSH/GSSG ratio decreased at the 3 and 6-hour time points, recovering to baseline by 24 hours in the sensitive group **(A)**. No significant change in GSH/GSSG ratio was seen in the resistant group **(B)**. Data presented as mean ± SEM (n=8; *p<0.05, compared with control). A single color was used in all figures to represent a single donor.

GSH/GSSG ratio was measured following 1, 3, 6 and 24-hour exposure to 1 mM H_2_O_2_ in the basal chamber (Figure 3). In the sensitive group (Figure 3A), GSH/GSSG ratio dropped significantly at 3 and 6-hours (*p*=0.024, *p*=0.018, respectively), with recovery observed by 24-hours. These data indicate the epithelium was able to recover from OS by 24 hours, presumably as the H_2_O_2_ stimulus had dissipated. In the resistant group (Figure 3B), two donors had reduction in GSH/GSSG ratio at 3-hours, overall, there was no significant differences in GSH/GSSG ratio at any time point (*p*=0.533).

### H_2_O_2_ exposure decreased cellular ATP

Most of the intracellular ATP is produced in mitochondria. ATP production is an indicator of mitochondrial health. A decline in mitochondrial function can cause a decrease in ATP production [25].

**Figure 4.**
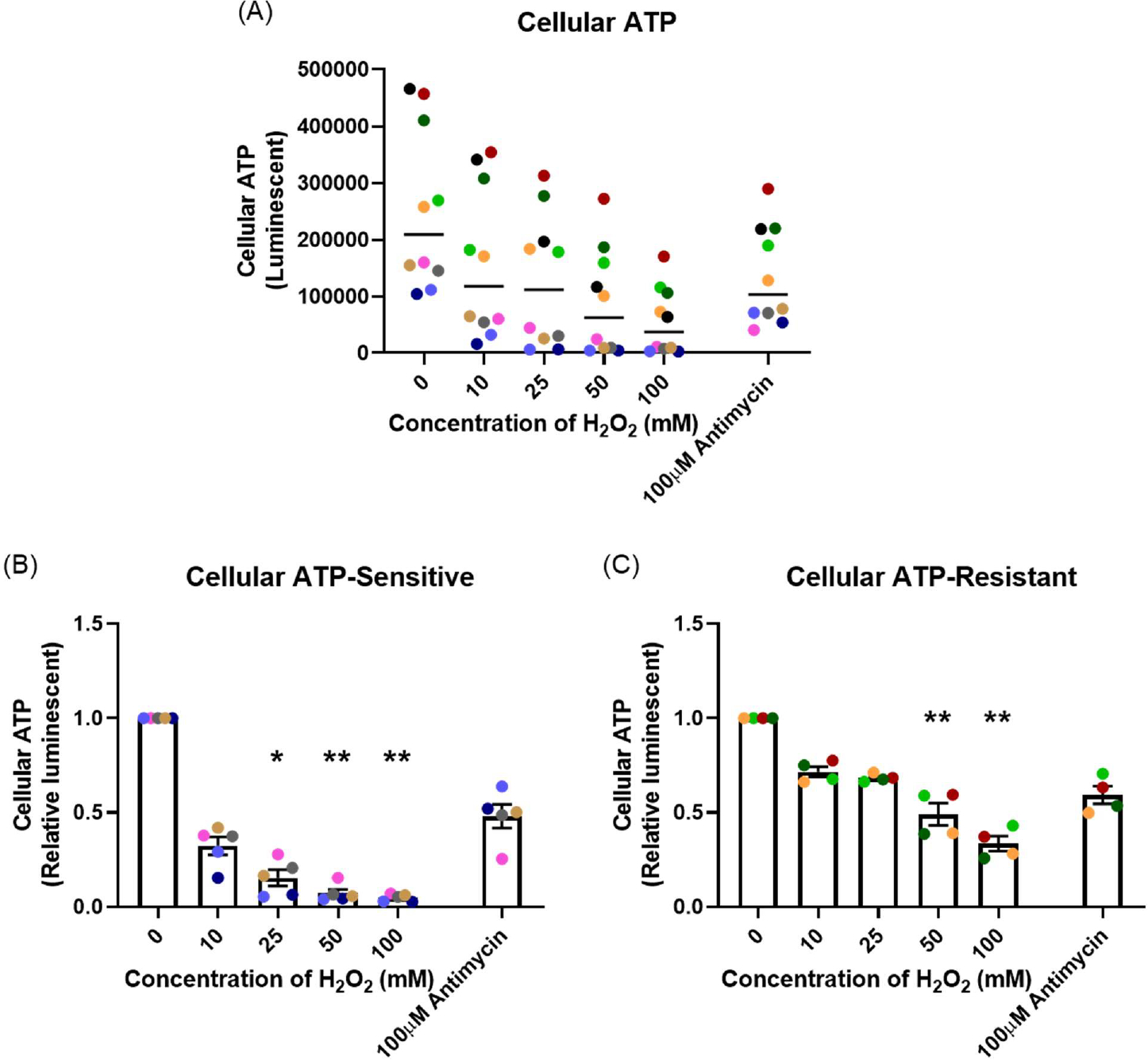
The effect of oxidant exposure on mitochondrial ATP production, measured following one-hour H_2_O_2_ exposure. The resistant individuals had significantly higher ATP baseline compared with the sensitive individuals presented as raw luminescent readings **(A)**. There was a dose-dependent decrease in both sensitive **(B)** and resistant **(C)** groups. Data presented as mean ± SEM (n=10; *p<0.05; ** *p*<0.01, compared with control). A single color was used in all figures to represent a single donor.

After one-hour H_2_O_2_ exposure, ATP production was measured. Figure 4A shows the data as the raw luminescence reading. Interestingly, the resistant donors had much higher cellular ATP baseline compared with the sensitive donors (*p*=0.008). A dose-dependent decrease in cellular ATP in both sensitive (*p*=0.001; Figure 4B) and resistant (*p*=0.004; Figure 4C) groups were observed. The sensitive donors decreased ATP production to a much greater degree than the resistant donors at the same concentration of H_2_O_2_ (*p*<0.001). Antimycin (100 μM), an inhibitor of complex III in mitochondrial electron transport chain [25], was included as positive control. Antimycin induced a reduction in ATP production of the same magnitude in both groups (*p*=0.008, *p*=0.032, respectively). Data in Figures 4B and 4C are shown normalized to the baseline ATP concentration.

### Stress response signaling pathway

To determine changes in the SIRT1-mediated signaling pathways following oxidant exposure, mRNA expression (*SIRT1*, *p53*, *p21*, *FOXO3*, *PINK1*), protein expression (total and phosphorylated SIRT1 and PARKIN protein levels) and NRF2 nuclear translocation was examined.

#### mRNA expression:

**Figure 5.**
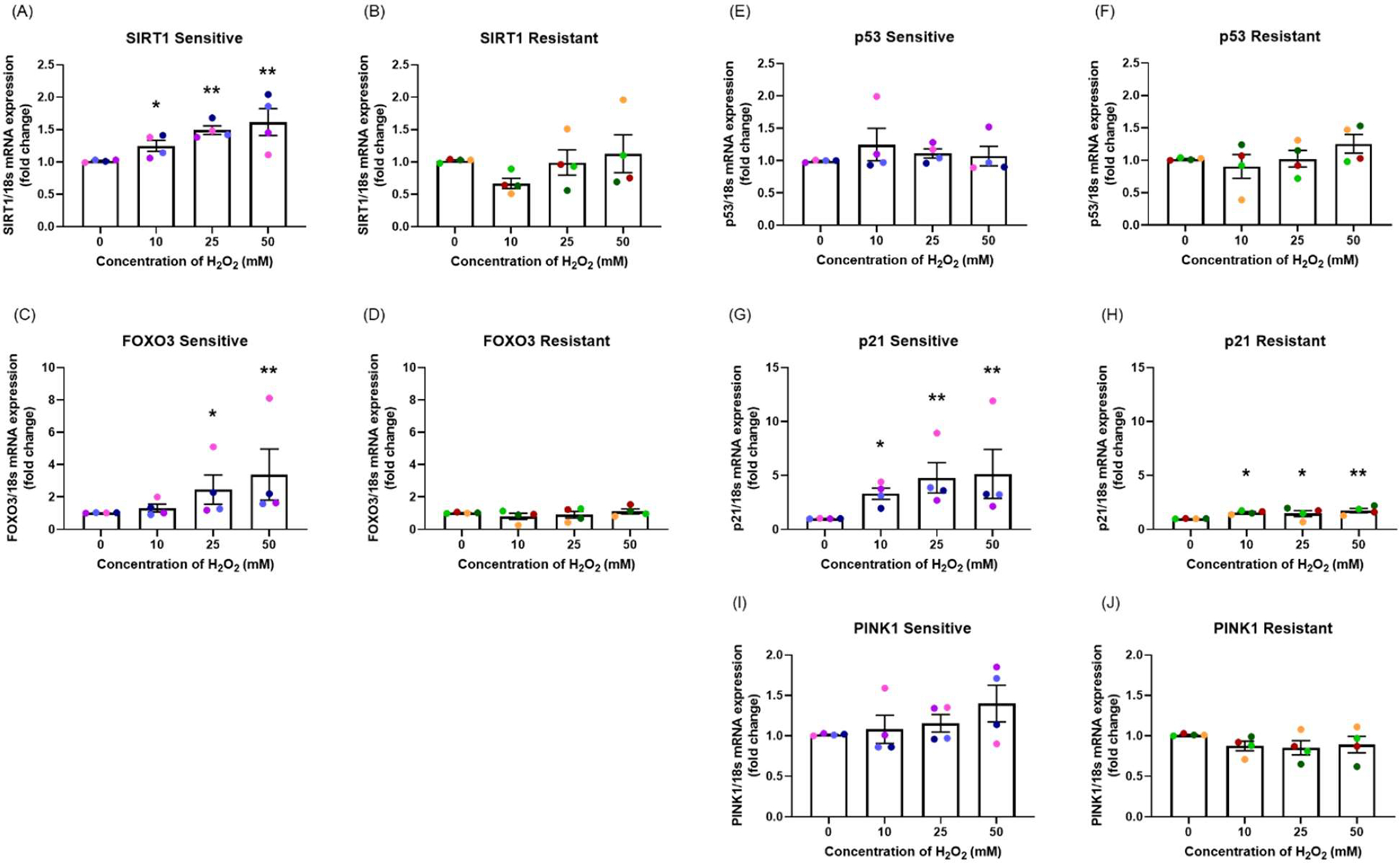
The effect of oxidant exposure on mRNA expression levels. Following H_2_O_2_ exposure, *SIRT1* **(A, B)**, *FOXO3* **(C, D)**, *p53* **(E, F)**, *p21* **(G, H)**, and *PINK1* **(I, J)** mRNA expression levels were measured. There was a dose-dependent increase in *SIRT1*, *FOXO3*, *p21*, and *PINK1* mRNA expression with no changes in *p53* mRNA expression in the sensitive group. In the resistant group, H_2_O_2_ exposure up-regulated only *p21* mRNA expression. There were significant differences between the groups in *SIRT1*, *FOXO3*, *p21*, and *PINK1* mRNA expression. Data presented as mean ± SEM (n=8; *p<0.05; ** *p*<0.01, compared with control). A single color was used in all figures to represent a single donor.

mRNA expression was measured at 24-hour following H_2_O_2_ exposure. In the sensitive group, there was a dose-dependent increase in *SIRT1* (*p*=0.015; Figure 5A), *FOXO3* (*p*=0.046; Figure 5C), and *p21* (*p*=0.033; Figure 5G), no change in *p53* mRNA expression (*p*=0.628; Figure 5E) and moderately increased *PINK1* mRNA expression (*p*=0.518; Figure 5I). In the resistant group, H_2_O_2_ exposure only caused a dose-dependent increase in *p21* mRNA expression (*p*=0.095; Figure 5H), with no significant differences in *SIRT1* (*p*=0.106; Figure 5B), *FOXO3* (*p*=0.718; Figure 5D), *p53* (*p*=0.613; Figure 5F), or *PINK1* (*p*=0.277; Figure 5J) mRNA expression.

The sensitive donors had significantly higher mRNA expression of *SIRT1* (*p*=0.001; Figures 5A and 5B), *FOXO3* (*p*=0.003; Figures 5C and 5D), *p21* (*p*=0.005; Figures 5G and 5H), and *PINK1* (*p*=0.023; Figure 5I and 5J) compared to resistant donors.

#### Protein expression:

**Figure 6.**
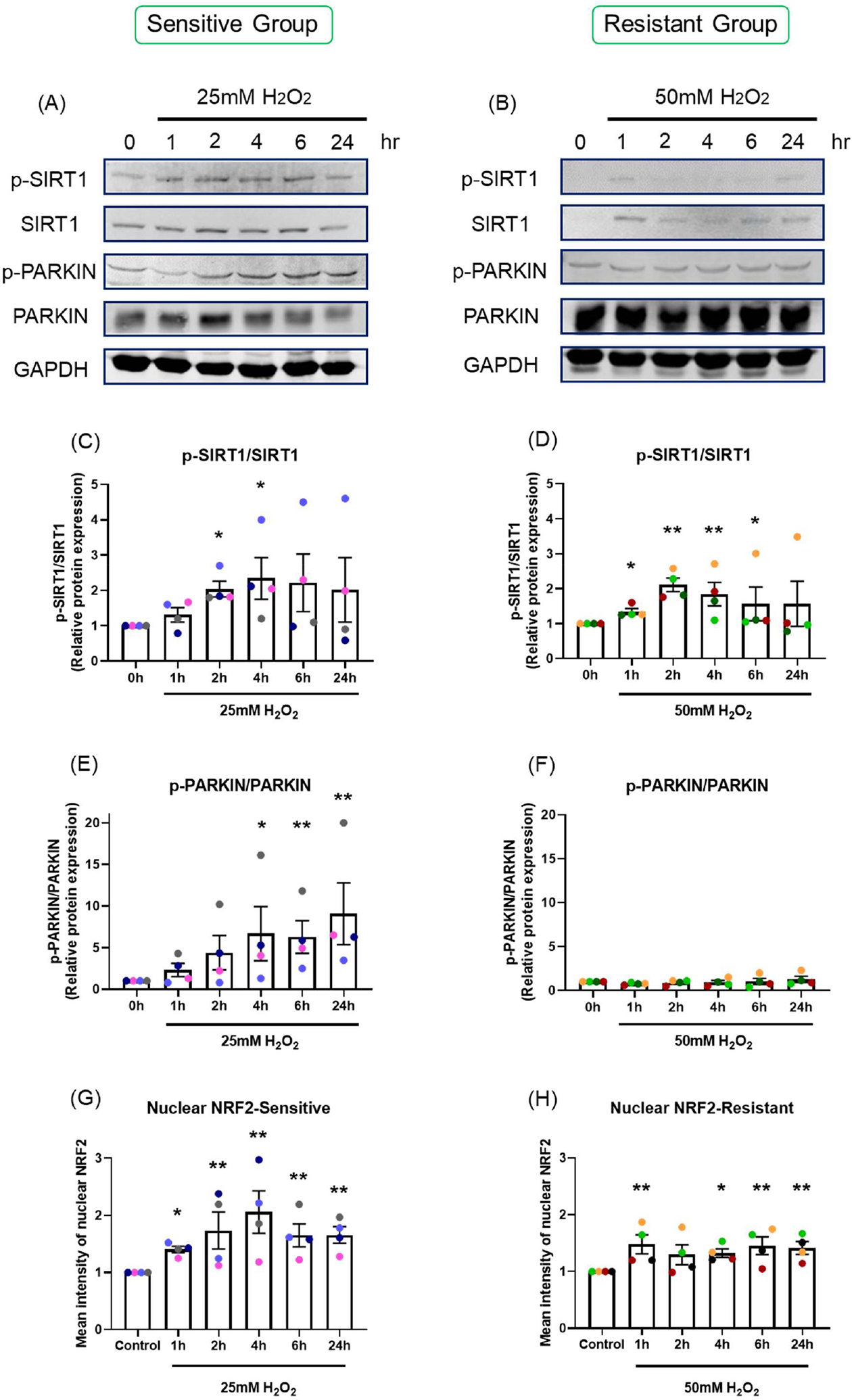
The effect of oxidant exposure on protein expression levels and nuclear NRF2 translocation. Following H_2_O_2_ exposure, SIRT1 and PARKIN phosphorylation was measured by western blot. Images are shown from representative sensitive **(A)** and resistant **(B)** donors. There was an increase in SIRT1 phosphorylation at 2 and 4-hour in both sensitive **(C)** and resistant groups **(D)**. PARKIN phosphorylation rose at later time point, 4 to 24-hour in the sensitive group **(E)**, but not in the resistant group **(F)**. The difference between the groups in p-PARKIN/PARKIN was *p*<0.001. Following one-hour H_2_O_2_ exposure confocal images were taken and nuclear NRF2 was quantified. NRF2 translocation was increased following H_2_O_2_ exposure and peaked at 4-hour in the sensitive group **(G)**. NRF2 translocation rose slightly in the resistant group **(H)**. Data presented as mean ± SEM (n=8; *p<0.05; ** *p*<0.01, compared with control). A single color was used in all figures to represent a single donor.

Protein phosphorylation was assessed by western blot at 1, 2, 4, 6, and 24-hour following H_2_O_2_ exposure. 50 μg of protein lysate was loaded in each lane (Figures 6A and 6B). Data are shown as the ratio of phosphorylated protein to total protein level (Figures 6C–6F). SIRT1 was maximally phosphorylated 2 to 4 hours after H_2_O_2_ exposure in both sensitive (*p*=0.022, *p*=0.015, respectively; Figure 6C) and resistant (*p*=0.002, *p*=0.007, respectively; Figure 6D) groups. PARKIN was phosphorylated at later time points, from 4 to 24-hour, in the sensitive group (*p*=0.018, *p*=0.008, *p*=0.002, respectively; Figure 6E). H_2_O_2_ exposure did not induce increases in phosphorylated or total PARKIN in the resistant group (*p*=0.498; Figure 6F). The sensitive donors had significantly higher PARKIN protein phosphorylation than the resistant donors (*p*<0.001; Figures 6E and 6F).

#### NRF2 translocation:

NRF2 translocation from the cytoplasm to the nucleus was assessed by immunofluorescence, with images captured by confocal microscopy (Figure S4), and nuclear NRF2 was quantified (Figures 6G and 6H).

Following H_2_O_2_ exposure, nuclear NRF2 increased and peaked at 4-hours in the sensitive group (*p*=0.046; Figure 6G). NRF2 translocation to the nucleus was less prominent in the resistant group, with only slight increases in nuclear NRF2 following H_2_O_2_ exposure (*p*=0.105; Figure 6H).

## Discussion

The impact of oxidant exposure and the mechanisms underlining OS in human airway epithelium is not fully understood. A decline in airway epithelial innate immune system functionality is associated with many medical conditions and can result in chronic inflammation and compromised immunity of the lung [26]. Most previous studies have been conducted on cell lines using submerged monolayer culture which directly exposes cells to H_2_O_2_ into the culture media [27]. Submerged monolayer epithelial cell cultures consists only of basal cells. The human airway epithelium contains only around 30% of basal cells [28,29]. 2D monoculture is fast and most of the existing assays have been optimised for monolayer culture, however, compared with 3D differentiated cells, the outcomes may not represent a true epithelial response [30]. In the present study, the effects of oxidant exposure on well-differentiated human airway epithelium cultured at ALI was investigated using various outcome variables. ALI cultures are a physiologically relevant model to human upper airways, the outcomes provide a better picture of the true epithelial response [19].

Firstly, epithelial integrity was measured following H_2_O_2_ exposure. There was a reduction in TEER and a rise in permeability and cell death in a dose-dependent manner. Decreased TEER and increased permeability indicate tight junction damage. This damage impairs the epithelial barrier, weakens epithelial defense, and allows greater access of environmental stressors to sub-epithelial structures [18].

Donor-to-donor variation in response to H_2_O_2_ was seen. Two clear groups were observed with different dose-response curves in the response of TEER and epithelial permeability to H_2_O_2_. For this reason, there was a need to understand how each donor responded and how those differences should be interpreted. Individual susceptibility to environmental oxidant exposures has been recognized. Epidemiological data have shown that African Americans have lower levels of plasma glutathione compared to Caucasians [31]. Genetic diversity in different ethnicities [32] and single nucleotide polymorphisms in particular genes in different races have been demonstrated to be related with higher risk of developing several diseases such as cancer [33]. Thus, ethnicity may be a factor that drives susceptibility to OS. However, in the present study, most of the donors were Caucasian and there was no difference in ethnicity between the sensitive and resistant groups. Some other factors such as sex, age, smoking history, socioeconomic status, education, have been considered, but there was no significant difference in background between the sensitive and resistant individuals. This might be because of the small sample size used here. A larger sample size in future studies may help to identify factors predicting the variability in response to oxidant stimuli.

Reactive oxygen species produced by mitochondria include free radicals such as hydroxyl radicals (•OH) and superoxide anions (O_2_•^−^) and nonradicals such as H_2_O_2_ [34]. Increased mtROS can damage mitochondrial DNA, depolarize mitochondrial membranes resulting in reduced membrane potential, impair electron transport, and lead to mitochondrial dysfunction [35]. In the present study, exogenous H_2_O_2_ exposure significantly increased mtROS generation. This result is consistent with a study in cartilage endplate cell showed that H_2_O_2_ induced mtROS production [36].

Acute oxidant exposure can induce mitochondrial hormesis (mitohormesis), a concept first described in 2006 [37]. Mitohormesis describes a process where sublethal mitochondrial stress induces antioxidant enzymes and enhances redox homeostasis [38,39]. Redox homeostasis is essential for antioxidant responses and maintenance of cellular health [40]. Modest increases in ROS can be detoxified by antioxidant enzymes such as catalase and SODs [41]. There are several ways to measure redox status including the ratio of reduced to oxidized glutathione (GSH/GSSG). Glutathione is the major intracellular antioxidant buffer, with a concentration between 2 to 5mM [42]. Glutathione peroxidase (GPx) catalyses H_2_O_2_ reduction via H_2_O_2_ + 2GSH to GSSG + 2H_2_O, a reaction that can be reversed by glutathione reductase (GR) [43]. Our data show an initial reduction in GSH/GSSG ratio 3 to 6-hours after H_2_O_2_ exposure, with recovery by 24 hours in sensitive donors. This showed that sensitive donors were initially susceptible to OS. The recovery by 24 hours could be due to effective mitohormesis or to the oxidant stressor being consumed. In either case, our data suggest that antioxidant defenses of the epithelium in sensitive donors could cope with a short-term exposure. The epithelial GSH/GSSG ratio from resistant donors was not influenced by H_2_O_2_ exposure, demonstrating resistance to induction of OS.

The majority of ATP is synthesized by F_1_F_0_ATP synthase (complex V) in the mitochondrial electron transport chain [44]. Protons generated by complexes I, III, and IV drive oxidative phosphorylation of ADP to ATP [25]. Decreased mitochondrial membrane potential can cause a reduction in ATP production. Our data show that H_2_O_2_ exposure caused a dose-dependent decrease in cellular ATP. The level of reduction was greater in the sensitive individuals than in the resistant individuals. The most interesting aspect of this result, seen in Figure 4A, was the different baseline levels of cellular ATP between the groups. The resistant individuals had much higher cellular ATP baseline than the sensitive individuals. The ATP concentration is significantly different within different cell types [45], but there is no literature showing differences in cellular ATP levels between individual humans or other living organisms. Our hypothesis is that the resistant individuals have more mitochondria or a greater ability to generate ATP compared with the sensitive individuals. Since mitochondria are involved in cellular metabolic processes and redox regulation [39], this phenomenon might be the key reason for the variability of OS susceptibility that was found in the present study. However, this needs further investigation.

SIRT1 plays an important role in mediating redox signaling [46]. The SIRT1-FOXO3 pathway can enhance activity of antioxidant enzymes, MnSOD and catalase, and promote DNA damage repair [47]. In the present study, H_2_O_2_ exposure caused a dose-dependent increase in *SIRT1* and *FOXO3* mRNA expression in the sensitive group, but not in the resistant group. Up-regulation of *SIRT1*-*FOXO3* signaling pathway indicated that the cells were trying to enhance antioxidant enzyme activity to prevent OS. This is in agreement with a previous study in renal tubular cells, where up-regulated *SIRT1*-*FOXO3* expression protected cells from H_2_O_2_ induced apoptosis [48].

Oxidant exposure can induce p53-p21 apoptosis, cell cycle arrest and cellular senescence related pathways [49]. In the present study, H_2_O_2_ exposure did not change *p53* mRNA expression and significantly increased *p21* mRNA expression. This indicated that the oxidant induced increase in *p21* might be regulated by a *p53* independent pathway, such as PI3K-AKT-p21 pathway [50].

The SIRT1-NRF2 pathway is associated with enhancing the antioxidant enzymes such as glutathione peroxidase [51]. Our data show that H_2_O_2_ exposure upregulated *SIRT1* mRNA expression, enhanced SIRT1 phosphorylation, NRF2 translocation at approximately 2 to 4-hour, and a recovery in the GSH/GSSG ratio at 24-hour was observed in the sensitive group.

The PINK1-PARKIN complex mediates mitophagy [52]. OS is associated with upregulation of the SIRT1-NRF2-PINK1-PARKIN mitophagy pathway to remove damaged mitochondria [12,53]. In the present study, H_2_O_2_ exposure induced *PINK1* mRNA upregulation and PARKIN phosphorylation at around 4 to 24-hour, occurring later that up-regulation of SIRT1 and NRF2 nuclear translocation.

The logistics of using primary cells grown into a well-differentiated respiratory epithelium dictated a relatively small sample size compared with cell lines and rodent models. The complexity of the studies limited the numbers of samples that could be included. An increased sample size will be required in future studies to fully explore this phenomenon. Differential responses to oxidant exposure between healthy individuals were demonstrated in the present study. This raises the question of how to identify and protect vulnerable members of the population from environmentally induced OS. A comprehensive survey in a large population may identify factors predisposing individuals to OS.

**Figure 7.**
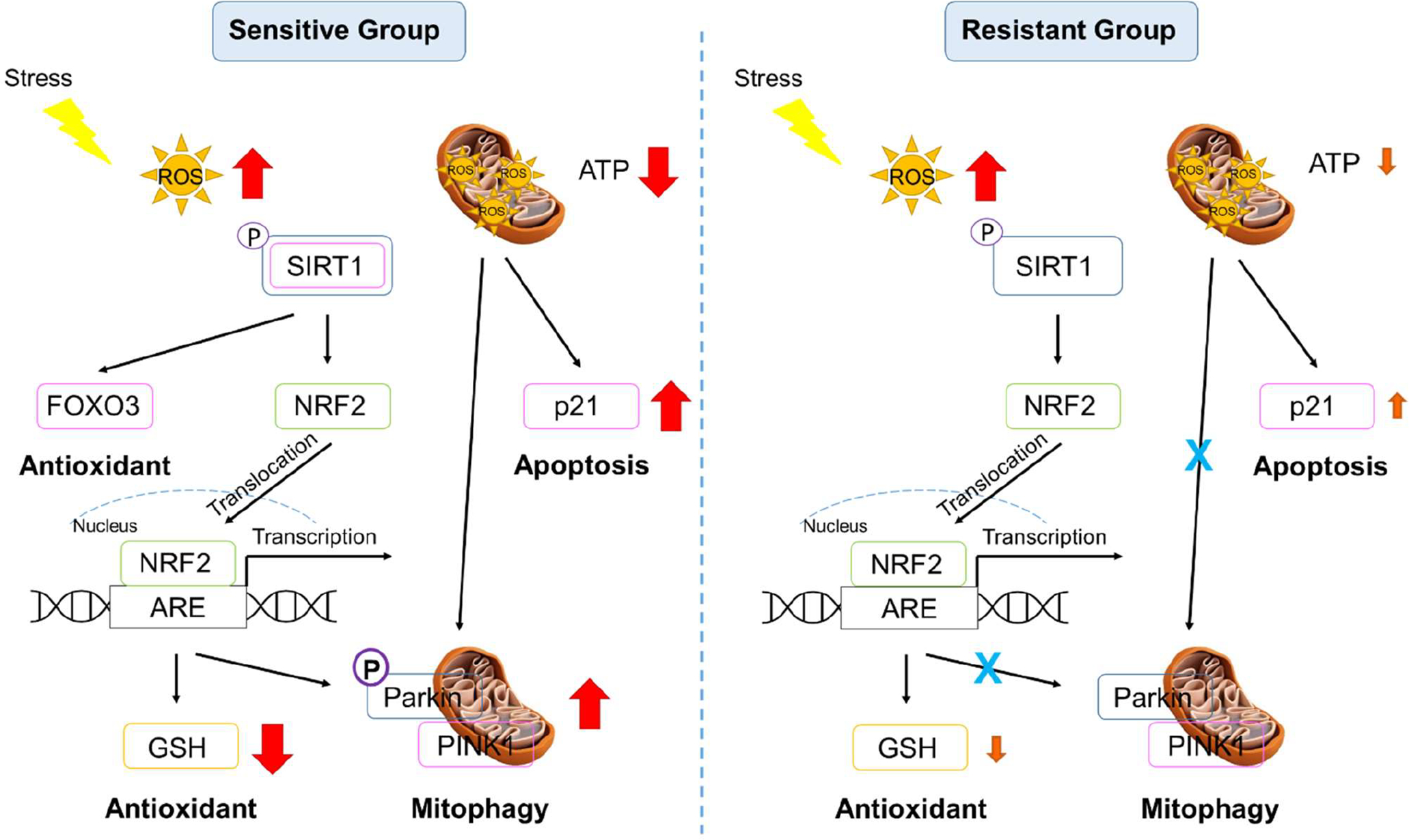
Signaling pathway diagram in the sensitive and resistant groups. Red: significant increase or decrease; Orange: mild increase or decrease.

In summary, the adverse impact of oxidant exposure on cellular and mitochondrial health were discussed in this study. The data presented have demonstrated the significant differences between healthy individual humans with differing susceptibility to OS. Major differences were shown in basal mitochondrial ATP production, activation of the *p21* apoptosis pathway, and upregulation of the PINK-PARKIN complex mitophagy pathway. Differences were also shown in mitohormesis with different responses in GSH/GSSG balance following oxidant exposure. The differences between individuals in susceptibility to OS may be attributed to resistant donors having more mitochondria or greater mitochondrial function. These cellular differences may account for the susceptibility of individual humans to environmental stressors. A diagram summarizing the differential pathways between the sensitive and the resistant individuals is shown in Figure 7.

## Supplementary Material

JCS-22-083_Supplementary File
