# Supplementary material for: Redox Homeostasis in Well-differentiated Primary Human Nasal Epithelial Cells
Source: J Cell Signal. Author manuscript; Available in PMC 2023 Feb 10. (PMC9912202; doi:10.33696/signaling.3.083)
Supplement: JCS-22-083_Supplementary File [file NIHMS1869065-supplement-JCS-22-083_Supplementary_File.pdf]

**Supplementary figures and tables**

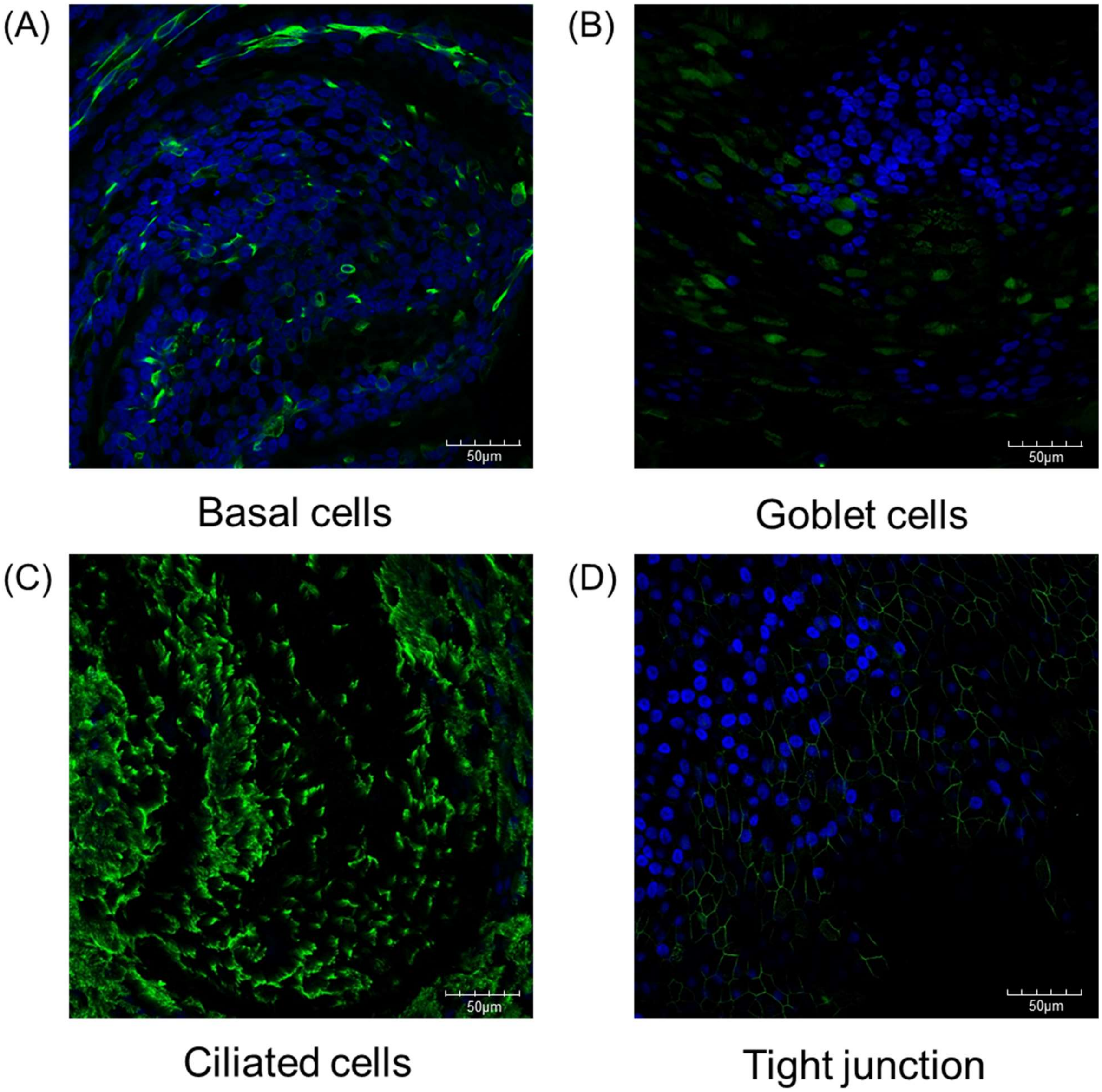


**Figure S1. Images present the cell types in differentiated epithelia cultured at ALI.** Immunostaining was used to detect basal cells, CK5 (A), goblet cells, MUC5B (B), ciliated cells, acetyl-α-tubulin (C) and tight junction, ZO-1/TJP1 (D). (Green indicates each cell type; Blue: Hoechst 33342 staining the nuclei). The scale bars represent 50μm.

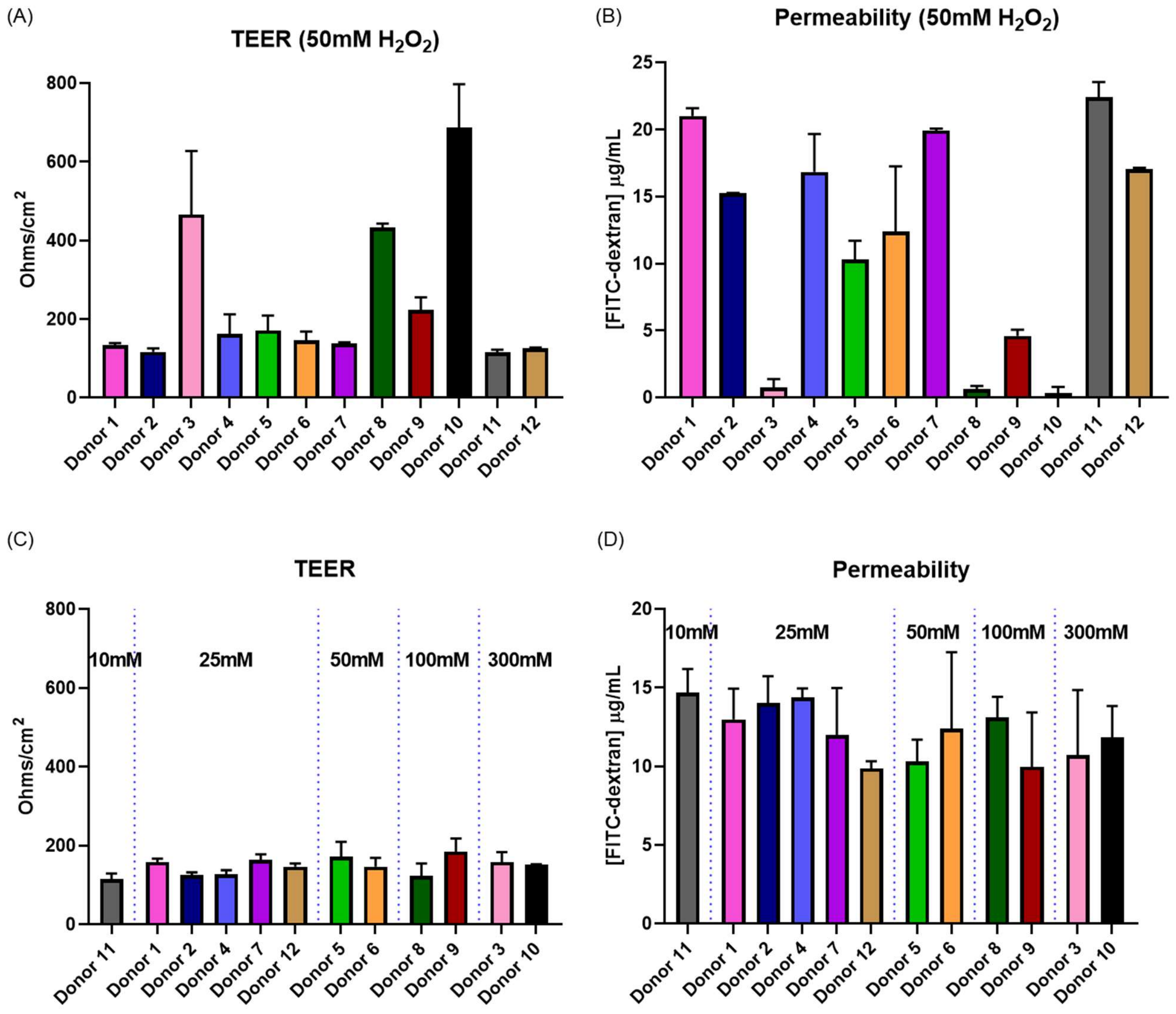


**Figure S2. Donor variability as seen in TEER and permeability measurements.** A permeability assay and TEER measurements were performed at 4-hour post 50mM $H_2O_2$ exposure and reproducible donor to donor variability were observed in response to oxidant exposure (A, B). Different $H_2O_2$ concentrations were used in each donor to achieve comparable baseline responses (C, D). The $H_2O_2$ concentration for each donor was chosen when the FITC-dextran concentration reaches 10 µg/ml in permeability assay.

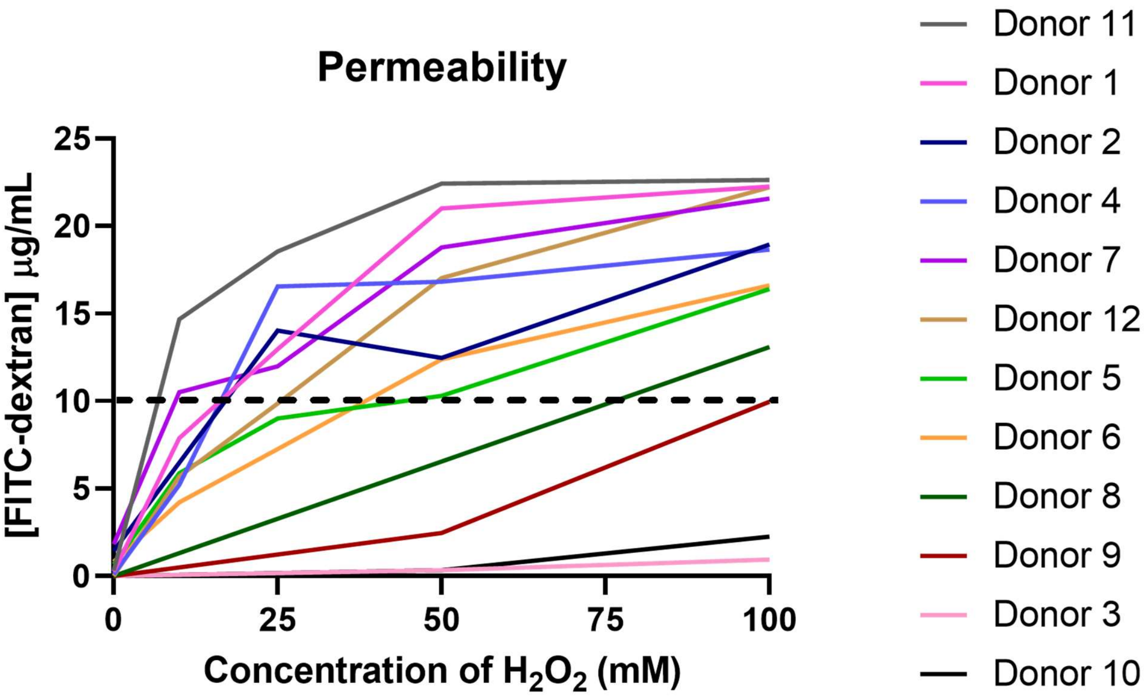


**Figure S3. Individual $H_2O_2$ dose-response in permeability.** The $H_2O_2$ concentration that caused the FITC-dextran concentration measured greater than 10 µg/ml was selected as the $H_2O_2$ exposure concentration. 25mM $H_2O_2$ was used in the top 6 donors (sensitive) and 50mM $H_2O_2$ was used in the bottom 6 donors (resistant). These concentrations were sublethal and were able to investigate the cellular responses.

| | Sex | Age | Ethnicity | Education level | Smoking status |
|---|---|---|---|---|---|
| **Sensitive** | | | | | |
| Donor 11 | M | 24 | Caucasian | Bachelor | no |
| Donor 1 | F | 38 | Caucasian | Doctoral | no |
| Donor 2 | M | 38 | Asian | Doctoral | no |
| Donor 4 | M | 27 | Caucasian | Bachelor | no |
| Donor 7 | F | 26 | Asian | Bachelor | no |
| Donor 12 | F | 40 | Caucasian | Bachelor | no |
| **Resistant** | | | | | |
| Donor 5 | F | 30 | Caucasian | Doctoral | no |
| Donor 6 | F | 31 | Caucasian | Bachelor | quit for 9 years |
| Donor 8 | M | 22 | Caucasian | Bachelor | quit for 2 years |
| Donor 9 | F | 21 | Caucasian | Bachelor | No |
| Donor 3 | F | 26 | Caucasian | Bachelor | no |
| Donor 10 | F | 25 | Caucasian | Bachelor | no |

**Table S1. Donor background**

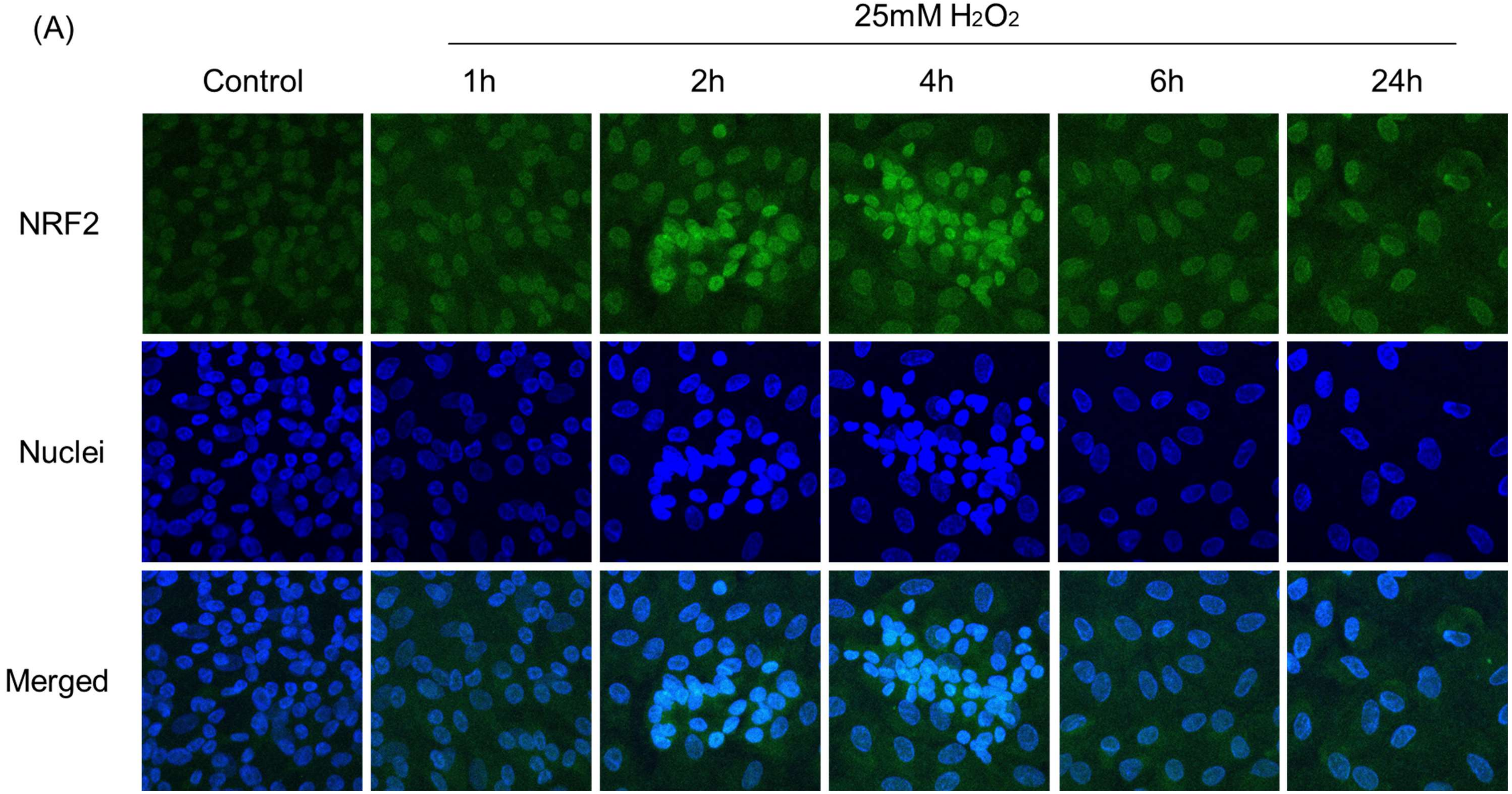

(A)
25mM H2O2
Control
1h
2h
4h
6h
24h
NRF2
Nuclei
Merged

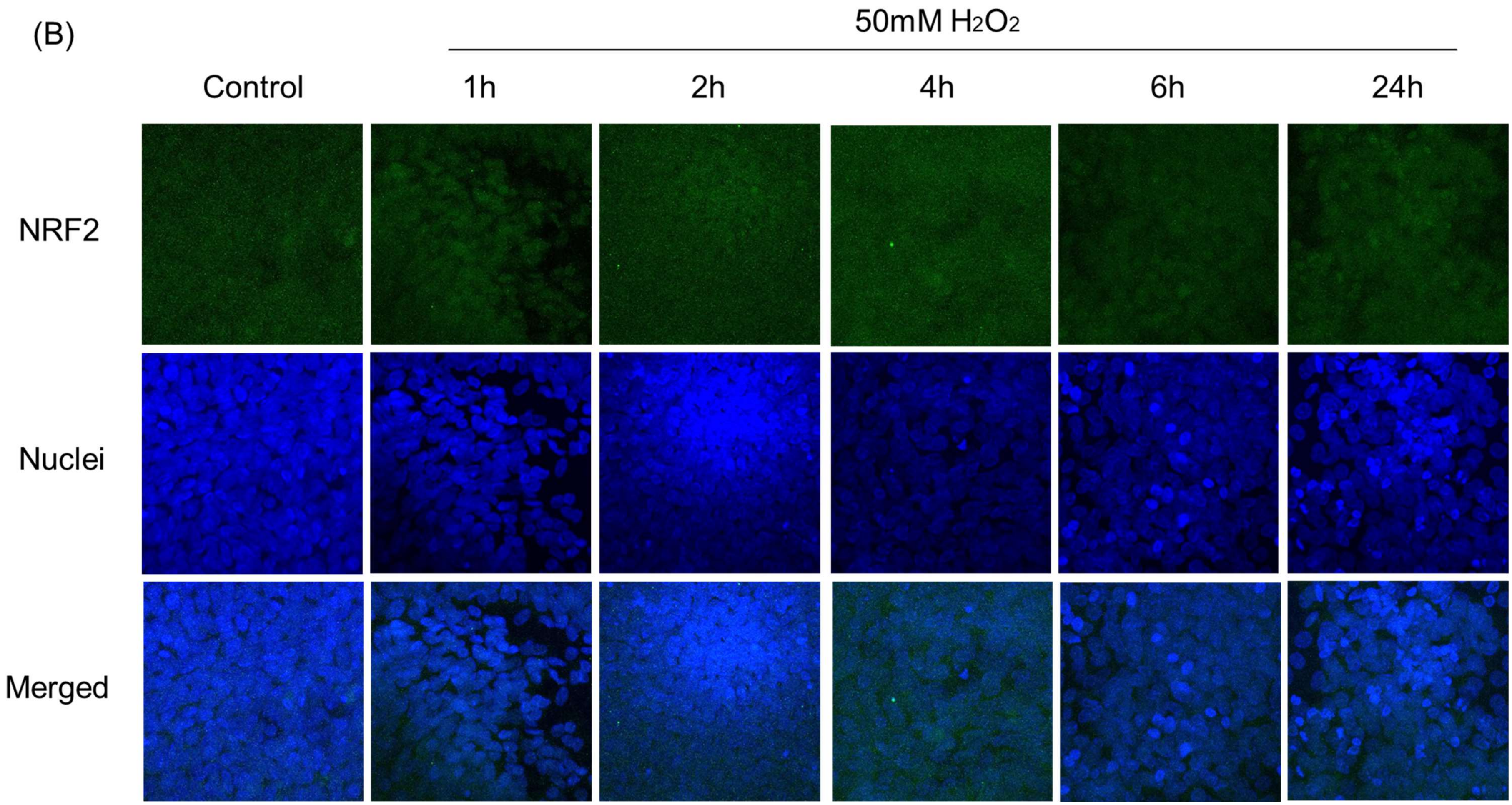

(B)
50mM $H_2O_2$
Control
1h
2h
4h
6h
24h
NRF2
Nuclei
Merged

**Figure S4. The effect of oxidant exposure on nuclear NRF2 translocation.** Following one-hour $H_2O_2$ exposure confocal images were taken. Images of a representative sensitive individual (A) and of a representative resistant individual (B). Grouped data are shown in Figure 6G, H.
